# Supplementary material for: Dissecting mechanisms of ligand binding and conformational changes in the glutamine-binding protein
Source: eLife. 2026 Jun 2;13:RP95304. doi: 10.7554/eLife.95304 (PMC13229503; doi:10.7554/eLife.95304)

Figure 2-source data 1. PDF file containing SDS page gel for Figure 2A, indicating the relevant bands and treatments.

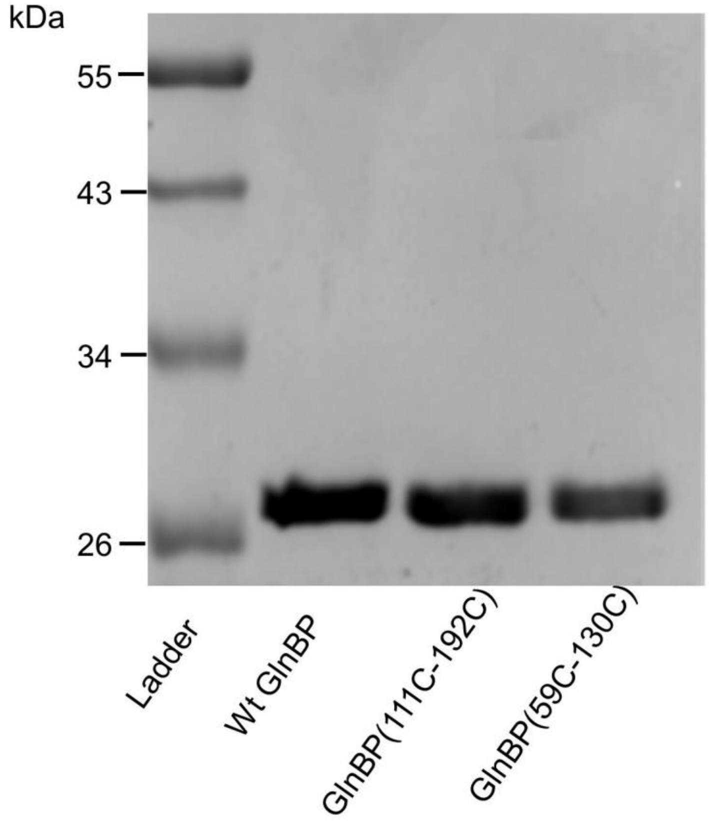

Supplement: Figure 2—source data 1. [file elife-95304-fig2-data1.pdf]
