# Supplementary figures and images for: Dissecting mechanisms of ligand binding and conformational changes in the glutamine-binding protein
Source: eLife. 2026 Jun 2;13:RP95304. doi: 10.7554/eLife.95304 (PMC13229503; doi:10.7554/eLife.95304)

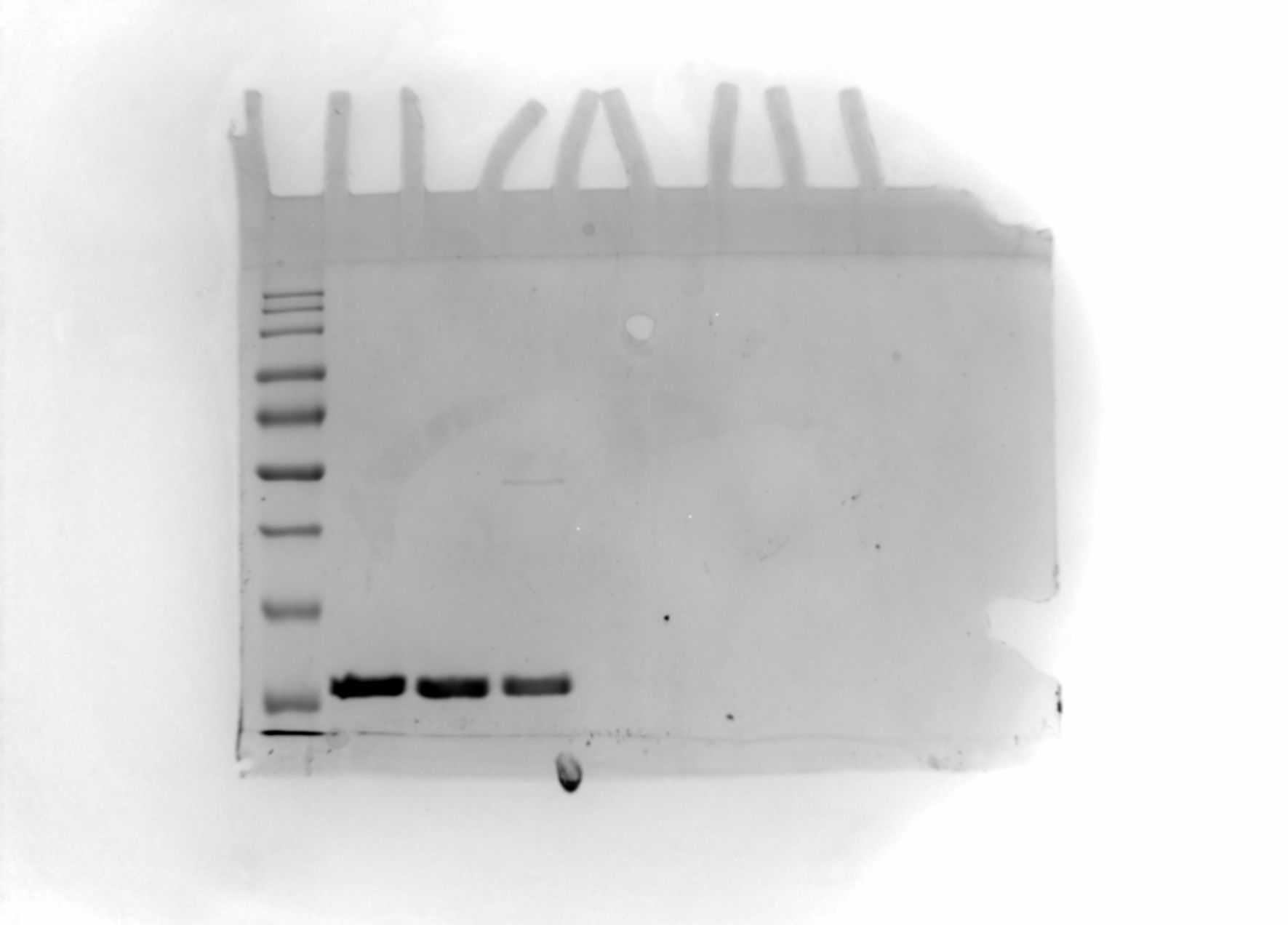

Supplement: Figure 2—source data 2. [file elife-95304-fig2-data2.zip › Figure 2-source data 2.jpg]
